# MGMIN: A Normalization Method for Correcting Probe Design Bias in Illumina Infinium HumanMethylation450 BeadChips

**DOI:** 10.3389/fgene.2020.538492

**Published:** 2020-10-27

**Authors:** Zhenxing Wang, Yongzhuang Liu, Yadong Wang

**Affiliations:** School of Computer Science and Technology, Harbin Institute of Technology, Harbin, China

**Keywords:** DNA methylation, design bias, normalization, M-value, Gaussian mixture model, Illumina Infinium 450K

## Abstract

The Illumina Infinium HumanMethylation450 Beadchips have been widely utilized in epigenome-wide association studies (EWAS). However, the existing two types of probes (type I and type II), with the distribution of measurements of probes and dynamic range different, may bias downstream analyses. Here, we propose a method, MGMIN (*M*-values Gaussian-MIxture Normalization), to correct the probe designs based on *M*-values of DNA methylation. Our strategy includes fitting Gaussian mixture distributions to type I and type II probes separately, a transformation of *M*-values into quantiles and finally a dilation transformation based on *M*-values of DNA methylation to maintain the continuity of the data. Our method is validated on several public datasets on reducing probe design bias, reducing the technical variation and improving the ability to find biologically differential methylation signals. The results show that MGMIN achieves competitive performances compared to BMIQ which is a well-known normalization method on β-values of DNA methylation.

## 1. Introduction

DNA methylation, as a well-known epigenetic marker, plays an essential role in biological processes and complex genetic diseases like cancer and diabetes (Irizarry et al., 2009; Paul et al., 2016). The Illumina Infinium HumanMethylation450 (450K) BeadChip (Bibikova et al., 2011) provides measurements of the level of methylation at over 480K CpG sites and has been widely used in epigenome-wide association studies (EWAS) and large-scale projects, such as The Cancer Genome Atlas (TCGA). The probes in the Infinium 450K BeadChip come in two different designs, type I (*n* = 135,501) and type II (*n* = 350,076), in order to increase the genomic coverage of the assay. However, the methylation values (β-values or *M*-values) derived from the two types of designs exhibit different distributions. Particularly, the type I probes possess a larger range of measurement than the type II probes (Dedeurwaerder et al., 2011). The differences between the two types of probe designs may impact the downstream analyses.

Several approaches have been published to correct the probe design bias. A peak-based correction (PBC) method normalizes type II probes to render them comparable with type I probes (Dedeurwaerder et al., 2011). In fact, PBC gets poor performance when the density distribution of methylation values does not show well-defined peaks. SQN (Touleimat and Tost, 2012) and SWAN (Maksimovic et al., 2012) select subset of probes with similar biological category to adjust the probe design bias. Beta MIxture Quantile dilation (BMIQ) is a model-based normalization approach to correct β-values of type II probes according to the beta distribution of β-values of type I probes, which appears to outperform PBC, SQN, and SWAN (Teschendorff et al., 2012).

In this work, we propose a method to correct the probe design bias based on the Gaussian Mixture Model (GMM) of the *M*-values of DNA methylation, which is called *M*-value Gaussian-MIxture Normalization (MGMIN). The method includes three steps: (i) fit Gaussian-mixture distributions to type I and type II probes separately, (ii) utilize a transformation of *M*-values into quantiles, (iii) perform a dilation transformation based on *M*-values to maintain the continuity of the data. We evaluate MGMIN using several independent datasets in terms of reducing the replicate technical variance and correcting the type II bias. By comparison with BMIQ, the results show that MGMIN improves the overall performance of normalization.

## 2. Materials and Methods

### 2.1. Measure DNA Methylation With *M*-value

The β-value of DNA methylation for each probe is defined by the ratio of the methylated intensity (M) and the overall intensity (sum of methylated intensity and unmethylated intensity: M + U):

$$\beta -value=\frac{M}{M+U+\alpha }$$

where α is a constant offset (by default, α = 100) to regularize the β-value when the overall intensity is low. The β-value falls between 0 and 1 which follows a Beta distribution naturally. A β-value of 0 indicates the CpG site of the measured sample is fully unmethylated and a value of 1 indicates that the CpG site is completely methylated.

The *M*-value is calculated by the log2 ratio of the methylated intensity (M) vs. the unmethylated intensity (U):

$$M-value=\log_{2}\left(\frac{M+\alpha }{U+\alpha }\right)$$

where α here is also an offset (by default, α = 1) to counteract the big changes caused by small intensity estimation errors. An *M*-value close to zero indicates that the measured CpG site is about hemimethylated. A positive *M*-value suggests that more copies of the measured CpG site are methylated than unmethylated and a negative *M*-value means more copies of the CpG site are unmethylated. The *M*-value has been widely used in two-color expression microarray analysis (Du et al., 2010).

Due to more than 95% CpG sites have intensities more than 1,000 in Illumina methylation data, the α in β-value and *M*-value has an insignificant effect on observed results. So the relationship between β-value and *M*-value is shown as (with α ignored):

$$\beta =\frac{2^{M}}{2^{M}+1};M=\log_{2}\left(\frac{\beta }{1-\beta }\right)$$

According to the conclusions in Du et al. (2010), the *M*-value is more statistically valid in an analysis by modeling the distribution of *M*-values because of it's *homoscedastic*. So we choose to adjust the *M*-values of type II probes into the distribution property of type I probes to correct the probe design bias.

### 2.2. MGMIN: *M*-value Gaussian-MIxture Normalization

Gaussian Mixture Model (GMM) has been widely applied as a clustering method in analyzing gene-expression microarray data (Yeung et al., 2001; Pan et al., 2002) and used to detect differential gene expression (McLachlan et al., 2006). In this paper, we apply GMM to distinguish different methylation states of CpG sites for further correction. The *M*-values of a single 450K microarray can be viewed as a finite Gaussian mixture model of several methylation states (hypomethylated-U, hemimethylated-H, hypermethylated-F). The probability density function of the *M*-value for a single CpG site (*M*_*i*_) is defined as:

(1) $$\begin{array}{l} p\left(M_{i};\theta \right)=\overset{K}{\underset{k=1}{\sum }}\pi_{k}N\left(M_{i}|\mu_{k},\sigma_{k}^{2}\right) \end{array}$$

where *p*(*M*_*i*_, θ) represents the model density for *M*_*i*_ with unknown parameter vector θ, K is the number of different methylation states (components), $N\left(M_{i}|\mu_{k},\sigma_{k}^{2}\right)$ is the probability density function of the *k*th Gaussian component, and π_*k*_ is the mixing proportions which satisfy the constraint that $\overset{K}{\underset{k=1}{\sum }}\pi_{k}=1$ and 0 ≤ π_*k*_ ≤ 1. The parameter vector θ consists of the mixing proportions π_*k*_, the mean value μ_*k*_ and the standard deviation σ_*k*_, which can be estimated by the EM algorithm.

Next, we describe MGMIN in detail. First, *M*-values of type I and type II probes are modeled by GMM separately. Let $\mu_{T}^{S}$ and $\sigma_{T}^{S}$ denote the mean value and standard deviation where *S* ∈ (*U, H, F*) and *T* ∈ (*I, II*). *K*_*I*_ and *K*_*II*_ are the numbers of components for type I and type II probes, which are both set as 3 by default.

Second, each probe is assigned to hypomethylated (*U*_*T*_), hemimethylated (*H*_*T*_), or hypermethylated (*F*_*T*_) states by using the maximum probability criterion. Let $U_{T}^{L}$ ($U_{T}^{R}$) denote the *U*_*T*_ probes with *M*-values smaller (larger) than $\mu_{T}^{U}$, and let $F_{T}^{L}$ ($F_{T}^{R}$) represent the *F*_*T*_ probes with *M*-values smaller (larger) than $\mu_{T}^{F}$ where *T* ∈ (*I, II*). Then, we calculate the probabilities of $U_{II}^{L}$ probes, i.e.,

(2) $$\begin{array}{l} p=P\left(M_{U_{II}^{L}}|\mu_{II}^{U},{\left(\sigma_{II}^{U}\right)}^{2}\right) \end{array}$$

where P represents the cumulative distribution function of the Gaussian component. These probabilities are transformed back to quantiles (*M*-value) by using the parameters $\mu_{I}^{U}$ and $\sigma_{I}^{U}$ of type I probes, i.e.,

(3) $$\begin{array}{l} q=P^{-1}\left(p|\mu_{I}^{U},{\left(\sigma_{I}^{U}\right)}^{2}\right) \end{array}$$

where *P*^−1^ returns the value of the inverse cumulative density function given the probability p and q is the normalized *M*-values for $U_{II}^{L}$. The similar operation is performed on $F_{II}^{R}$ probes.

Then, we merge the $U_{II}^{R}$, *H*_*II*_, and $F_{II}^{L}$ probes into one set *G* on which a conformal (shift + dilation) transformation is performed. Some parameters are identified as *minG* = min{*M*_*G*_}, *maxG* = max{*M*_*G*_} and $\Delta_{G}^{M}=maxG-minG$. Similarly, the minimum value of $F_{II}^{R}$ and the maximum value of $U_{II}^{L}$ are also identified, i.e., $minF=\min\left\{F_{II}^{R}\right\}$ and $maxU=\max\left\{U_{II}^{L}\right\}$. Two distance values can be calculated as

$$\Delta_{UG}=minG-maxU$$

$$\Delta_{GF}=minF-maxG$$

The new normalized maximum and minimum values of G-probes are expected to satisfy the constraint that

$${maxG}^{\prime }=\min\left\{{F_{II}^{R}}^{\prime }\right\}-\Delta_{GF}$$

$${minG}^{\prime }=\max\left\{{U_{II}^{L}}^{\prime }\right\}+\Delta_{UG}$$

where ${F_{II}^{R}}^{\prime }$ and ${U_{II}^{L}}^{\prime }$ are new normalized values for $F_{II}^{R}$ and $U_{II}^{L}$, respectively. So the new normalized range value of set *G* is ${\Delta_{G}^{M}}^{\prime }={maxG}^{\prime }-{minG}^{\prime }$. The normalized *M*-values of set *G*, ${M_{G_{II}}}^{\prime }$, is calculated by

(4) $$\begin{array}{l} {M_{G_{II}}}^{\prime }={minG}^{\prime }+d_{f}\left(M_{G_{II}}-minG\right) \end{array}$$

where $d_{f}={\Delta_{G}^{M}}^{\prime }/\Delta_{G}^{M}$ is the dilation factor. So, the normalized *M*-values for type II probes consist of *q* for $U_{II}^{L}$, ${M_{G_{II}}}^{\prime }$, and *q* for $F_{II}^{R}$.

$$M_{II}{}^{\prime }=(q_{U_{II}^{L}},\;M_{G_{II}}{}^{\prime },q_{F_{II}^{R}})$$

Lastly, the normalized *M*-values are transformed to β-values.

There are some important points to notice: (i) the initial values for μ and σ in EM algorithm are set as (−4,0,4) and (1,1,1) and small perturbations to the initial μ and σ will not affect the final model because MGMIN captures the natural property of the *M*-value of DNA methylation, (ii) *K*_*I*_ will be changed to 4 automatically when $\mu_{I}^{F}-\sigma_{I}^{F}$ is smaller than $\mu_{II}^{F}-\sigma_{II}^{F}$ in order to ensure that $\mu_{I}^{F}$ can always be larger than $\mu_{II}^{F}$ and avoid the presence of an unexpected peak in transformed *M*-values of hypermethylated type II probes, (iii) if *K*_*I*_ = 4, the *F*_*I*_ will be the set of probes belonging to the component with the largest μ, while the *U*_*I*_ contains the probes belonging to the component with the smallest μ and the other two components are assigned to *H*_*I*_, (iv) no thresholds need to be set by default or estimated by manual to distinguish the three different states of DNA methylation.

### 2.3. Datasets

We selected several public 450K datasets as following:

Dataset 1: GSE29290 downloaded from GEO considered in Dedeurwaerder et al. (2011). We used the three replicates (GSM15136, GSM15137 and GSM15138) from the HCT116WT cell-line and matched bisulfite pyrosequencing (BPS) date for nine type II probes of sample GSM815138 (r3) (Table 1 in Dedeurwaerder et al., 2011) to evaluate the performance of different methods.

Dataset 2: GSE38268 downloaded from GEO considered in Lechner et al. (2013) which consists of 6 fresh frozen HNC samples. We selected 5 samples as same as (Teschendorff et al., 2012), of which 2 were HPV+ and 3 HPV− (GSM937820 to GSM937824).

Dataset 3: GSE38266 downloaded from GEO considered in Lechner et al. (2013) which contains 21 FFPE HPV+ HNSCC samples and 21 FFPE HPV− HNSCC samples. Note that the entire quality of the dataset GSE38266 is not high.

Dataset 4: GSE95036 downloaded from GEO considered in Degli Esposti et al. (2017) which contains 6 HPV+ HNC samples and 5 HPV− HNC samples.

## 3. Results

### 3.1. MGMIN Needs No Default Initial Values of Parameters

Similar to the mixture model of BMIQ, MGMIN applies Gaussian mixture models for *M*-values instead of beta-mixture models for β-values. MGMIN also uses quantile information to correct the *M*-values of the type II probes into a distribution which is comparable with that of type I probes. MGMIN complies the inherent Gaussian mixture distributions for *M*-values of type I and type II probes to avoid setting any parameters manually, which is different from the default breakpoints in BMIQ. Thus, MGMIN needs less manual intervention than BMIQ. However, MGMIN is slightly inferior to BMIQ on some dataset (Table 1) due to the entire low quality of the dataset. Note that the PPV of BMIQ on Dataset 3 is lower than that of no normalization (RAW).

**Table 1:** Comparison of MGMIN and BMIQ on detecting the differentially methylated probes (DMPs) associated with HPV status was performed by counting the number of DMPs (Dataset 2), the number of validated differentially methylated probes (nTPs) (Dataset 3: GSE38266 and Dataset 4: GSE95036) and corresponding estimates for the positive predictive value (PPV = nTP/nDMPs).

**Metric**	**Raw**	**BMIQ**	**MGMIN**
nDMP	51 (51^a^)	239 (252^a^)	220
nTP (GSE38266)	16 (13^a^)	55 (51^a^)	37
PPV (GSE38266)	0.31 (0.25^a^)	0.23 (0.20^a^)	0.17
nTP (GSE95036)	3	13	27
PPV (GSE95036)	0.06	0.05	0.12

a *Values reported in Teschendorff et al. (2012)*.

### 3.2. MGMIN Reduces Technical Variation

**Figure 1:**
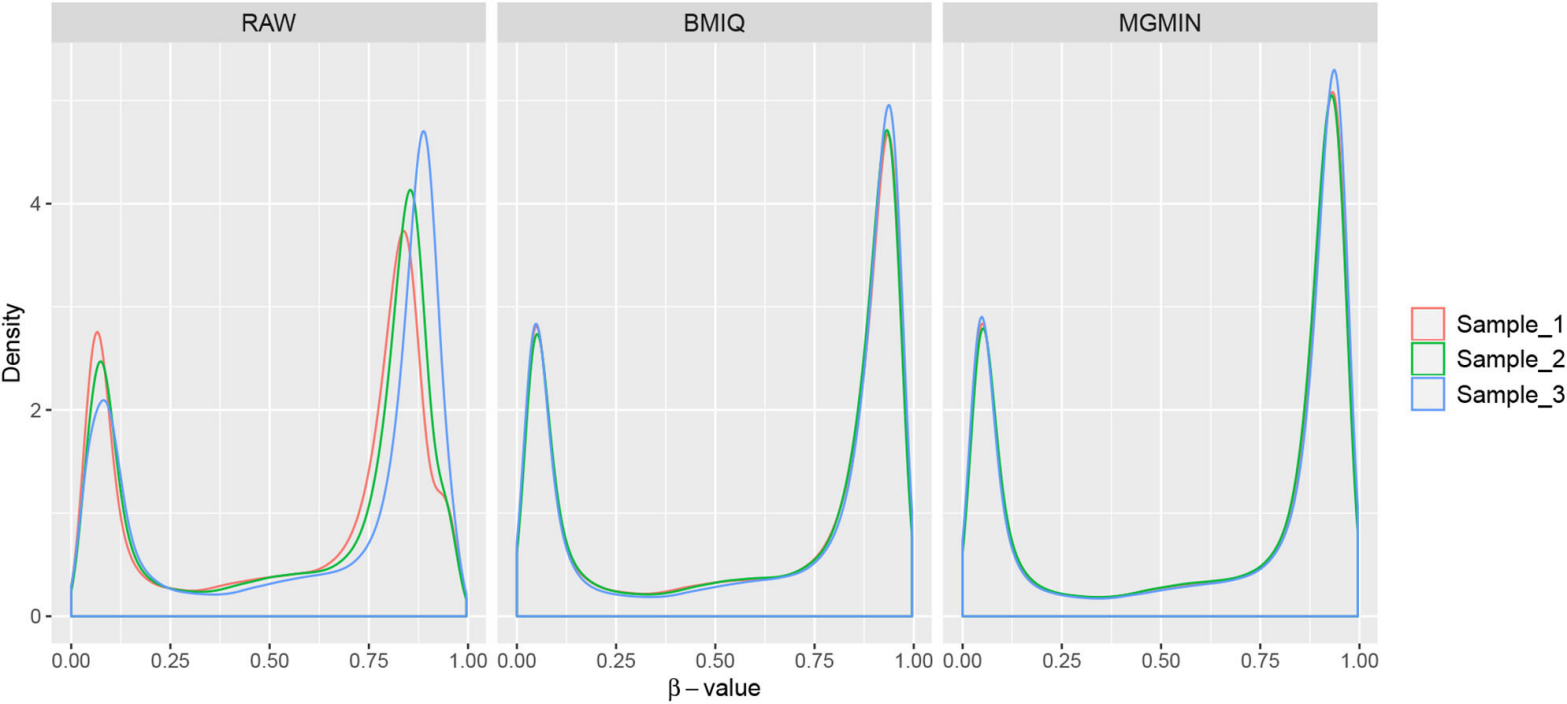
The density curves of β-values for the three replicates in Dataset 1. The left panel is for the case of raw data with no normalization, middle panel for BMIQ and right panel for MGMIN.

**Figure 2:**
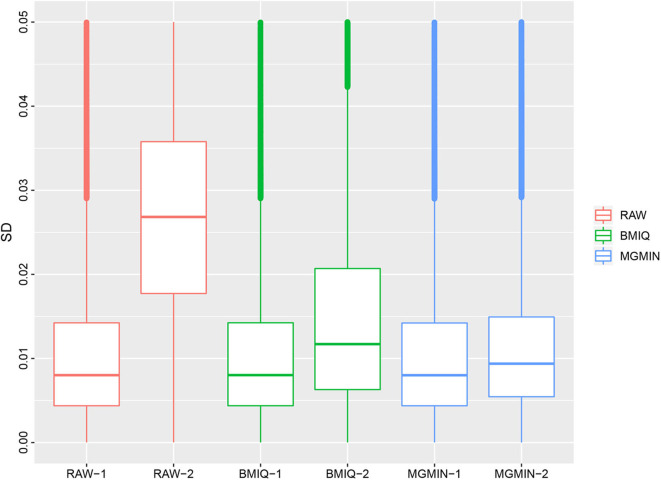
Boxplots of the standard deviations of β-values for the three replicates in Dataset 1, for raw β-values (RAW), normalized β-values by BMIQ (BMIQ), and normalized β-values by MGMIN (MGMIN). RAW-1 represents the type I of raw values and RAW-2 represents the type II of raw values, and so on.

**Figure 3:**
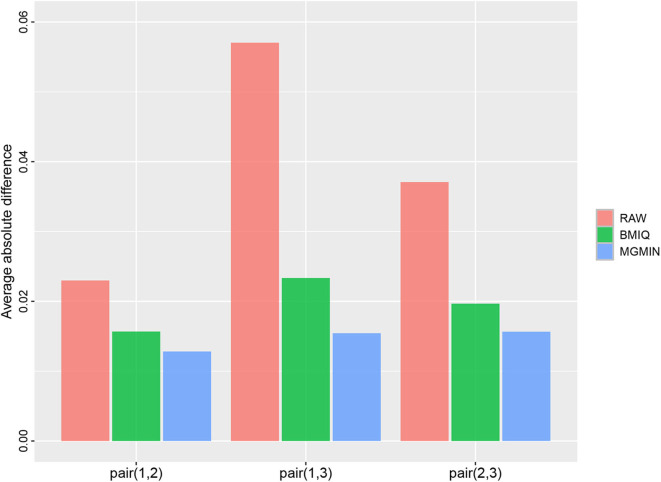
Barplots of the average absolute difference in β-values of type II probes between two samples in each of the three pairs of the three replicates in Dataset 1.

MGMIN is applied to Dataset 1 to identify the ability to improve reproducibility. The standard deviation (SD) for each probe across the three replicates was computed using no normalization (RAW), BMIQ, and MGMIN separately. As can be seen in Figure 1, both MGMIN and BMIQ almost made the density curves for the three replicates coincide with each other and reduced the technical variation significantly compared to no normalization. Compared to BMIQ, the standard deviation for type II probes adjusted by MGMIN is smaller (Figure 2). MGMIN also provided significant reduction of average absolute difference in β-values of type II probes between two samples in each of the three pairs of the three replicates (Figure 3).

### 3.3. MGMIN Reduces Probe Design Bias

**Figure 4:**
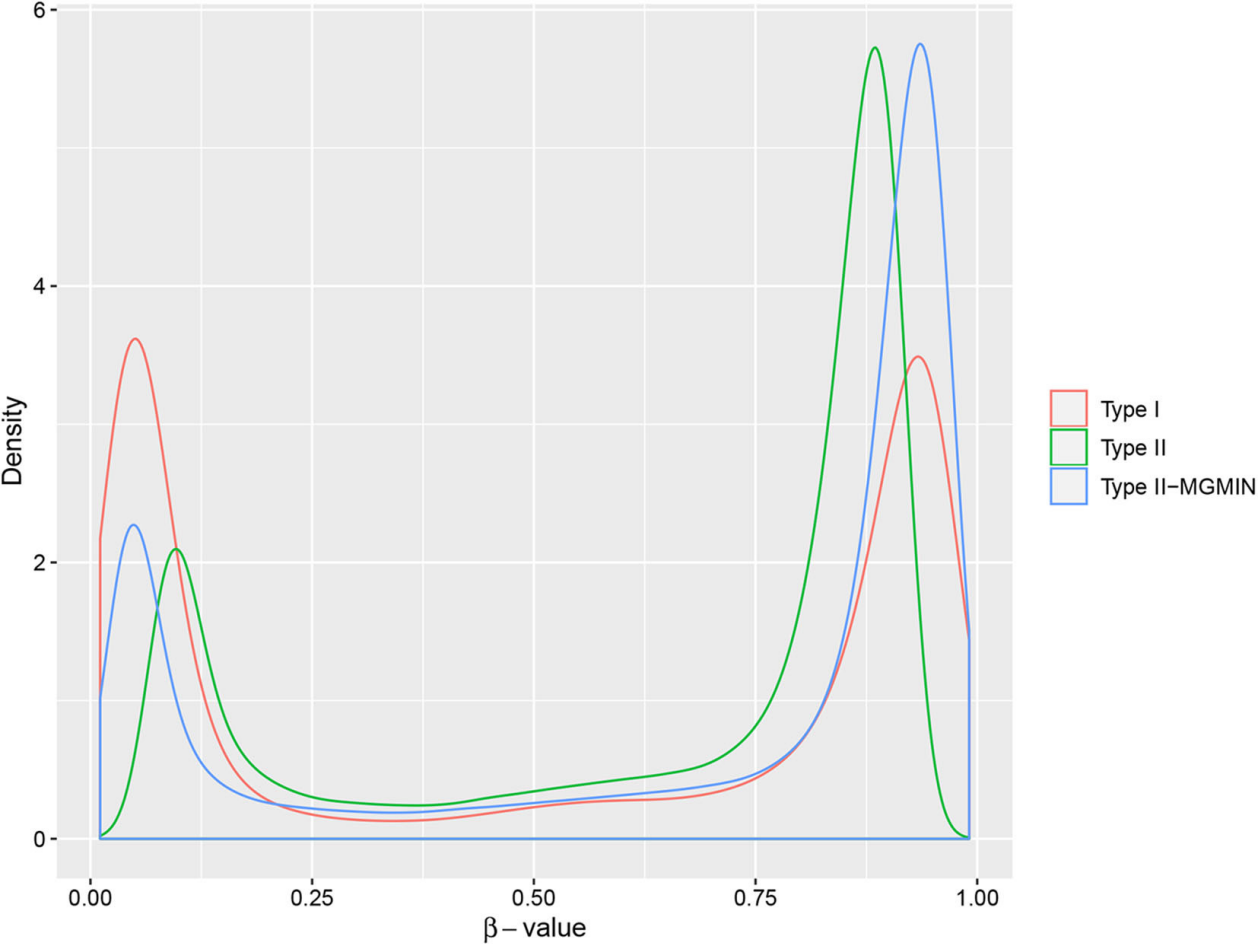
The density curves of β-values for type I probes, type II probes and normalized type II probes (type II-MGMIN) for sample GSM815138 from GEO29290.

**Figure 5:**
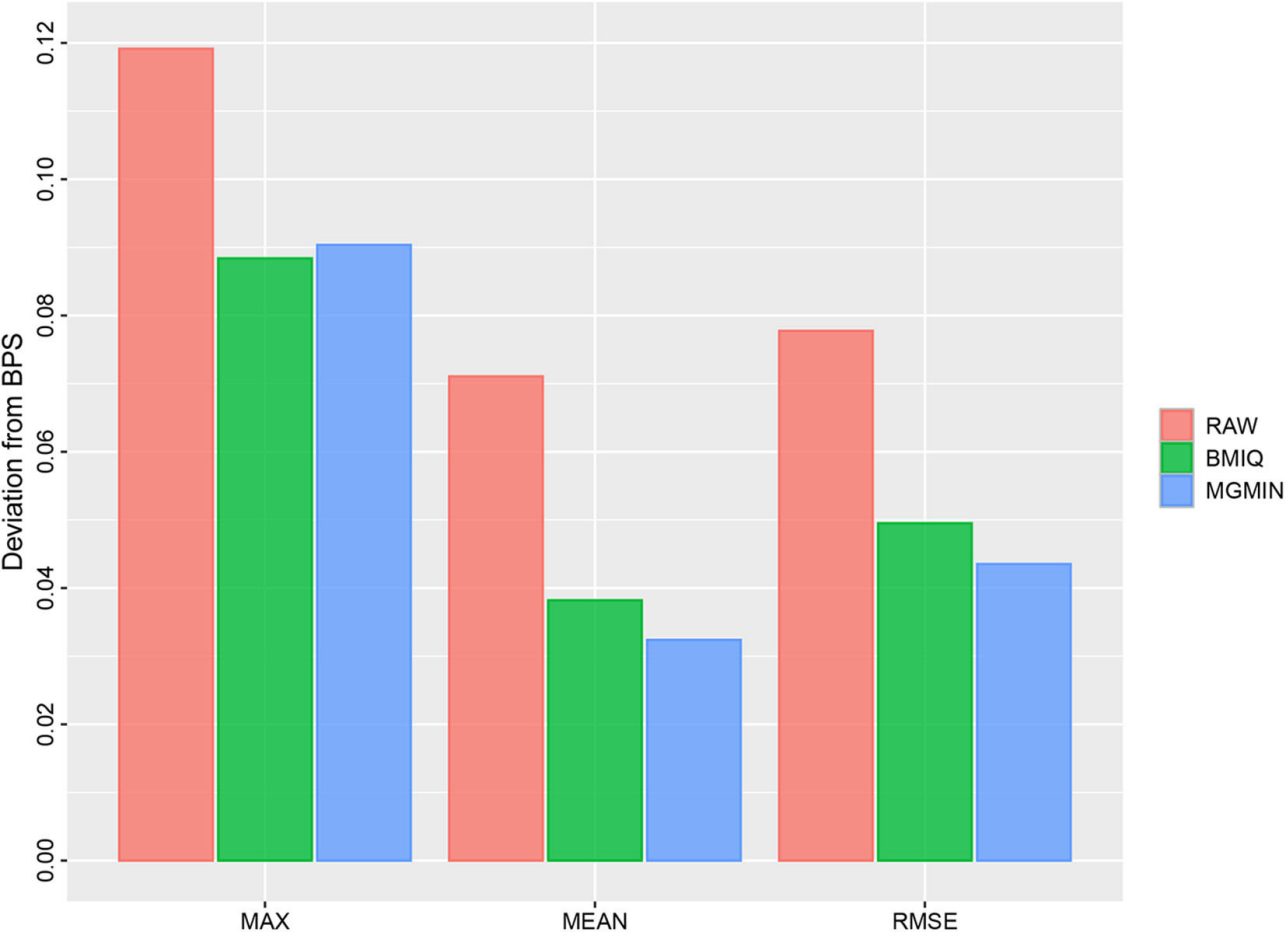
Barplots for the maximum (MAX), mean (MEAN) and root mean square error (RMSE) of the absolute deviation from the matched BPS values of nine type II probes for GSM815138 (r3) in Dataset 1 considered in Dedeurwaerder et al. (2011) using no normalization (RAW), BMIQ, and MGMIN, respectively.

MGMIN reduces the probe design bias via correcting the *M*-values of the type II probes such that the distribution curves for the *M*-values of the type I and type II probes show similar dynamic ranges and peaks (Figure 4). In Dedeurwaerder et al. (2011), the β-values for nine probes of type II by bisulfite pyrosequencing technique for sample GSM815138 (r3) were provided, which can be used as a gold-standard to evaluate the performance of different correction methods. Hence, we compared the normalized results of the nine type II probes in 450K arrays by MGMIN and BMIQ. As shown in Figure 5, although MGMIN performed slightly worse than BMIQ at the maximum value of the absolute deviation from BPS data, MGMIN significantly reduced the type II bias than BMIQ and raw data in terms of mean and root mean square error (RMSE) of the absolute deviation from the matched BPS values.

### 3.4. MGMIN Robustly Finds Informative Differential Methylation Probes Associated With HPV Status

**Figure 6:**
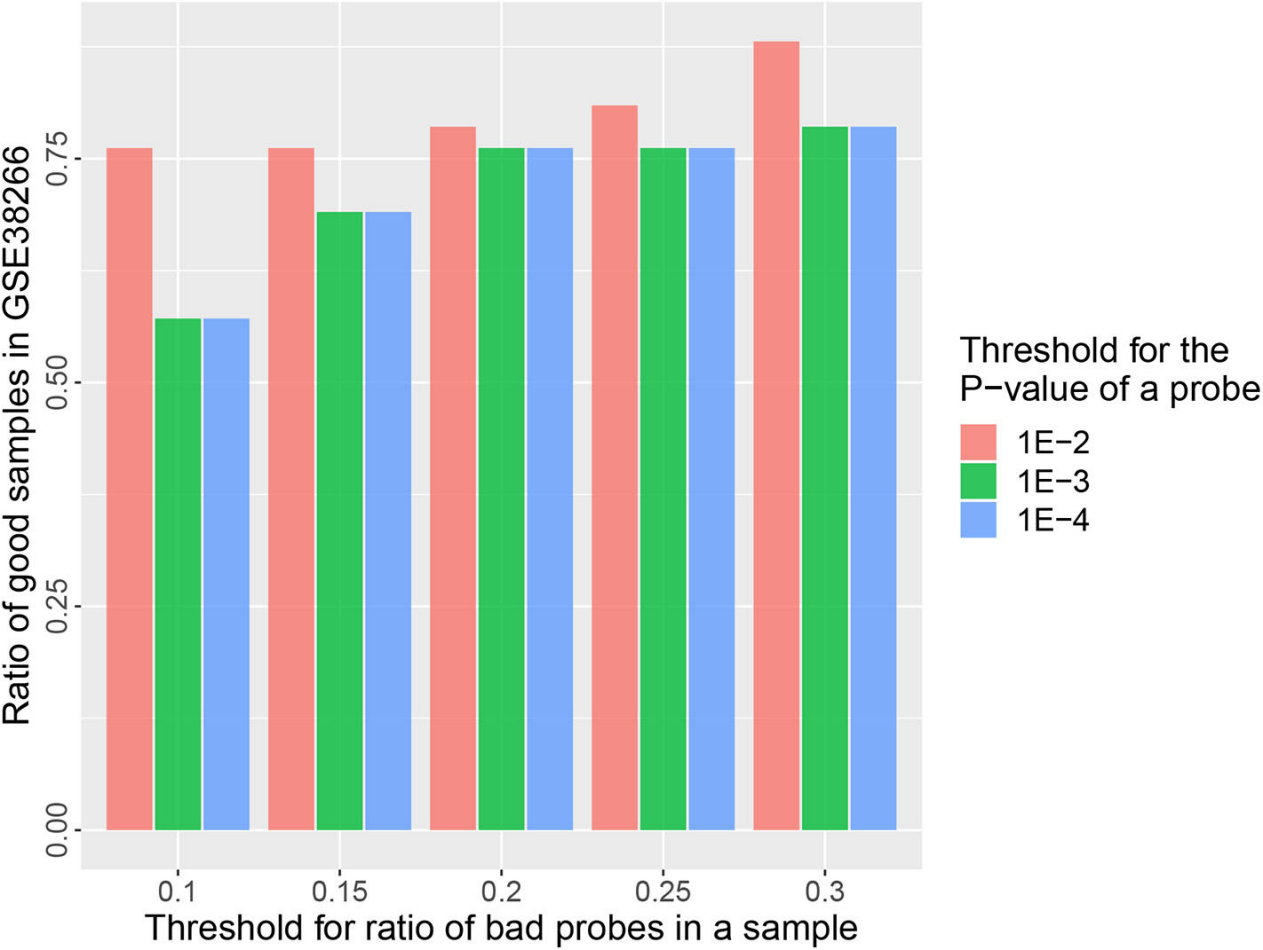
Barplots of the ratio of good samples in GSE38266 under different quality control options (τ_*p*_&τ_*r*_).

The goal of a bias correction approach is to reduce the technical variation and identify the biological informative signals at the same time. We used a strategy similar to Teschendorff et al. (2012) to compare the result between MGMIN and BMIQ in identifying the differential methylation probes (DMPs) associated with HPV status. First, Dataset 2 consisting of two HPV+ and three HPV− fresh frozen HNC samples were used as the training set to obtain the DMPs associated with HPV status by the *limma* method (Smyth, 2005) and an FDR threshold 0.35 which was as same as (Teschendorff et al., 2012). Both Dataset 3 and Dataset 4 described in the methods section were used as test set. We reanalyzed Dataset 2 and got similar numbers of DMPs to those reported in Teschendorff et al. (2012) with no normalization method (Raw) or BMIQ method (shown in Table 1). The results in Table 1 shows that the positive predictive value (PPV) of MGMIN is slightly less than BMIQ in terms of GSE38266 (Dataset 3) whereas MGMIN outperforms BMIQ in GSE95036 (Dataset 4). The reason for MGMIN slightly inferior to BMIQ in Dataset 3 may be the entire low quality of the dataset (see Figure 6) which is that the ratio of samples passing filters is <0.9 (*r* = 0.88) under the least restrictive condition. Let τ_*p*_ represent the *p*-value threshold for bad probes and τ_*r*_ represent the threshold for the ratio of bad probes in a sample. The maximum value of τ_*r*_ is set to 0.3 here in our opinion because a sample with more than 30% bad probes is vulnerable. We can get the same test dataset from GSE38266 with the one described in Teschendorff et al. (2012) which consists of 18 HPV+ and 14 HPV− samples under the following conditions: (i) τ_*p*_ = 1*e* − 4 or 1*e* − 3 and τ_*r*_ = 0.2 or 0.25, (ii) τ_*p*_ = 1*e* − 2 and τ_*r*_ = 0.1 or 0.15. Overall, MGMIN identified more true positive features than BMIQ.

## 4. Discussions

In this paper, we have proposed a method called MGMIN for correcting the probe design bias of type II probes in Illumina Infinium 450K BeadChips, which can reduce the technical variation and improve the ability to find biologically differential methylation signals. We have shown that MGMIN outperforms BMIQ on multiple evaluation datasets in correcting the type II design bias and improving the data quality.

Similar to BMIQ, MGMIN uses quantile information to correct the *M*-values of type II probes while leaving the *M*-values of type I probes unchanged. The three-state beta-mixture distribution model in BMIQ sets two default breakpoints (0.2, 0.75) to divide the β-values into three classes: hypomethylated, hemimethylated, and hypermethylated, which works well for most cases. However, the result curves of BMIQ show obviously inconsistent in some samples with high heterogeneity. We set 3 or 4 classes for probes depending on the result of $\mu_{T}^{F}-\sigma_{T}^{F}$ to ensure that the fitted hypermethylated component of type II probes can be located in the left of the hypermethylated component of type I probes, which can partly eliminate the effects of the heterogeneity of samples.

Based on the results of Dataset 3, we think the high quality of dataset is the base of normalization, in other words, there is no meaning to correct the samples with low quality. It should be pointed out that the parameter estimation of MGMIN is slower than that of BMIQ (about 1.5 times), which can be relieved by reducing the number of iterations.

MGMIN can be used in the 450K methylation data preprocessing with other methods to normalize the values of the two type probes and improve the data quality.

## Data Availability Statement

The datasets for this study can be found in GEO: GSE29290, GSE38268, GSE38266, and GSE95036.

## Author Contributions

ZW performed the experiments and wrote the manuscript. All authors read and revised the final manuscript.

## Conflict of Interest

The authors declare that the research was conducted in the absence of any commercial or financial relationships that could be construed as a potential conflict of interest.
